# Supplementary material for: Norovirus NS1/2 protein increases glutaminolysis for efficient viral replication
Source: bioRxiv. 2023 Dec 19:2023.12.19.572316. Preprint. [Version 1] doi: 10.1101/2023.12.19.572316 (PMC10769279; doi:10.1101/2023.12.19.572316)

Supplementary Figure 1:

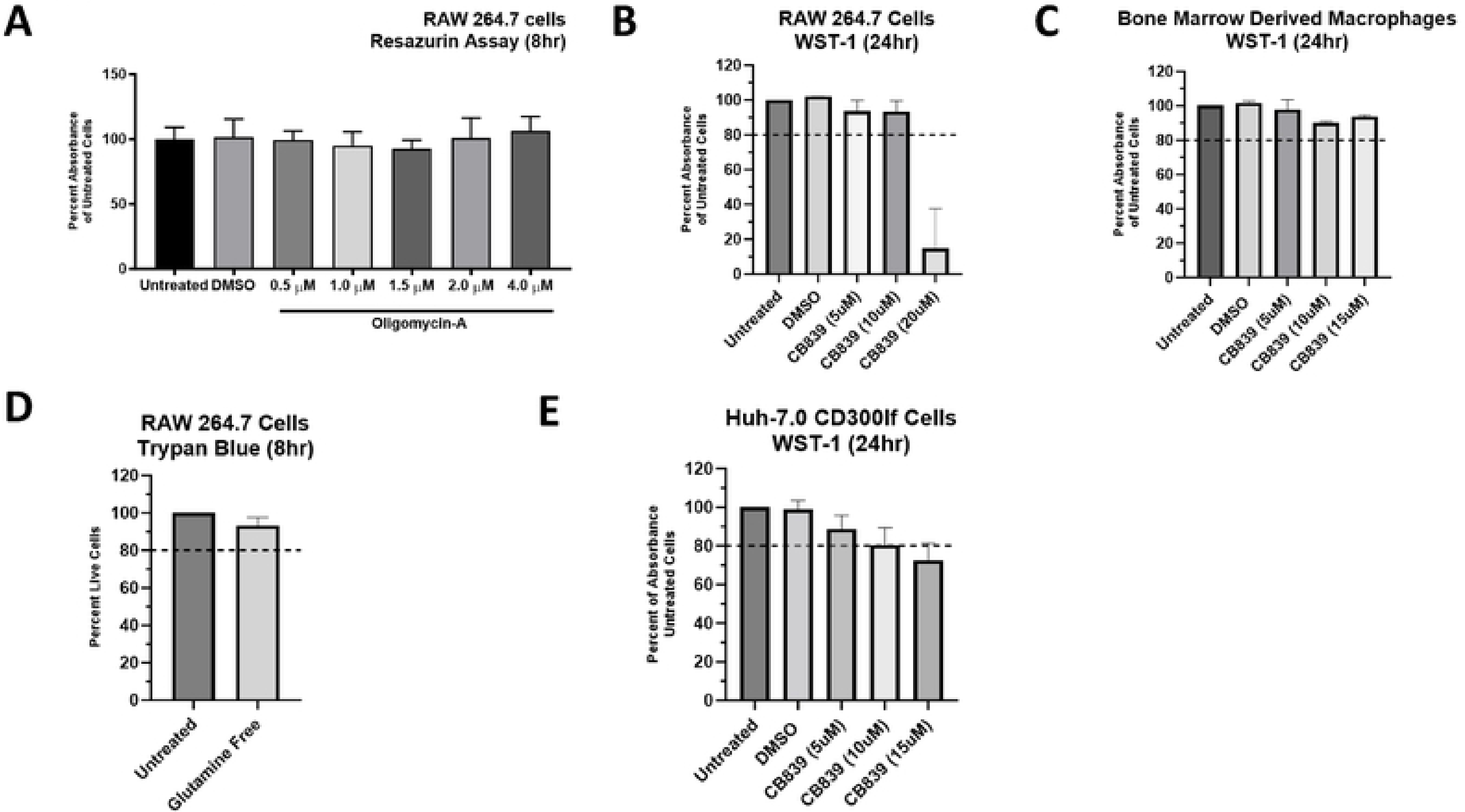

Supplementary Figure 2

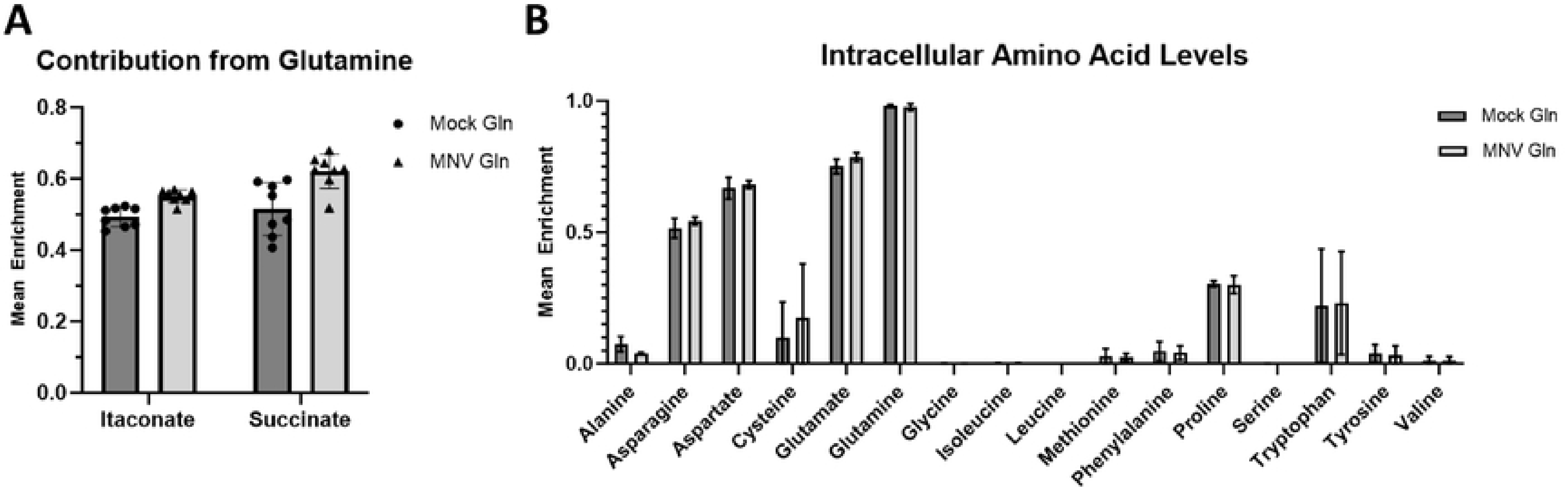

Supplementary Figure 3: Validation of MNV-1 non-structural protein expression

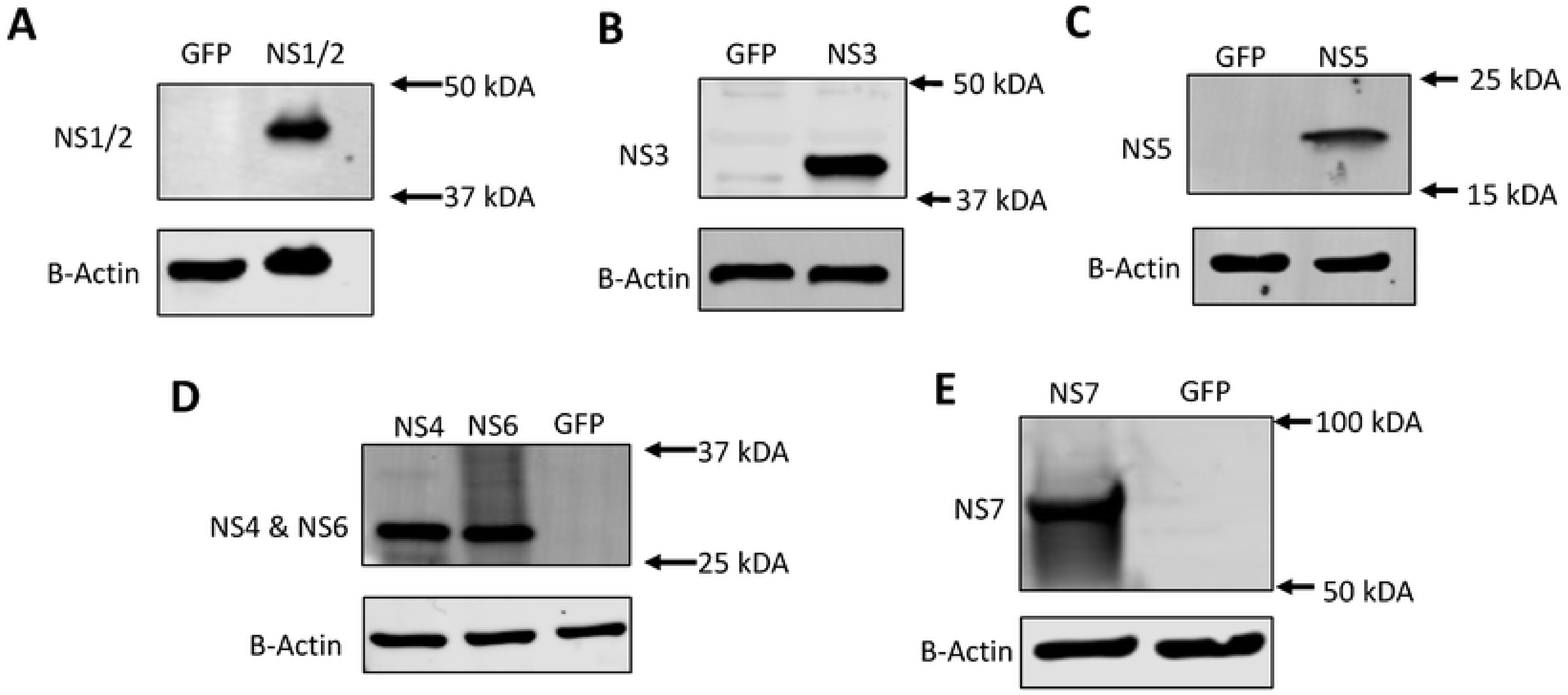

Supplement: Supplement 1 — Supplementary Figure 1: Cell viability assays of indicated cell lines. (A-B) RAW 264.7 cells were treated with indicated concentrations of (A) Oligomycin-A, (B) CB839, or vehicle control (DMSO) for either 8 or 24 hours, respectively. Cell viability was measured using Resazurin or WST-1 reagent. (C) Primary bone marrow-derived macrophages were treated with CB839 or vehicle control at the indicated concentrations for 24 hours. Cell viability was measured using WST-1 reagent. (D) RAW 264.7 cells were incubated with glutamine free or replete medium for 8 hours. Cell viability was measured using trypan blue staining on a Life Technologies Countess 3 automated cell counter assay platform. (E) Huh-7 CD300lf cells were treated with indicated concentrations of CB839 for 24 hrs. Cell viability was measured using WST-1 reagent. Experiments represent combined data from at least two independent experiments with two technical replicates each. Supplementary figure 2: MNV-1 infection does not alter the intracellular amino acid pool. (A-B) RAW 264.7 cells were either mock-infected or infected with MNV-1 for 1 hour at an MOI of 5. The virus inoculum was removed and replaced with medium containing 13C5-glutamine for 8 hours. Intracellular metabolites and amino acids were extracted with ice-cold methanol and measured by mass spectrometry. Experiments represent combined data from two independent experiments with four technical repeats. Successful expression of MNV viral proteins. Validation of MNV-1 nonstructural protein expression. (A-E) Huh-7 CD300lf cells were transfected with plasmids encoding the indicated MNV-1 nonstructural protein or green fluorescent protein. Transfected cells were incubated for 24–48 hours. Western blot analysis was performed to confirm successful expression. β-actin was used as a loading control. Data shows representative Western blots from 3 independent experiments. [file NIHPP2023.12.19.572316V1-supplement-1.pdf]
